# Trends in suicide rates during the COVID-19 pandemic restrictions in a major German city

**DOI:** 10.1017/S2045796021000019

**Published:** 2021-01-19

**Authors:** Daniel Radeloff, Rainer Papsdorf, Kirsten Uhlig, Andreas Vasilache, Karen Putnam, Kai von Klitzing

**Affiliations:** 1 Department of Child and Adolescent Psychiatry, Psychotherapy and Psychosomatics, University Hospital Leipzig, Leipzig, Germany; 2 Leipzig Health Authority, Leipzig, Germany; 3 Center for German and European Studies (CGES), Faculty of Sociology Bielefeld University, Bielefeld, Germany; 4 Department of Psychiatry, University of North Carolina at Chapel Hill, Chapel Hill, USA

**Keywords:** COVID-19, Germany, pandemic, quarantine, restrictions, suicide

## Abstract

**Aims:**

It remains unclear whether the coronavirus disease 2019 (COVID-19) pandemic is having an impact on suicide rates (SR). Economic insecurity and mental disorders are risk factors for suicide, which may increase during the pandemic.

**Methods:**

Data on suicide events in a major city in Germany, and the corresponding life years (LY) were provided by the local authorities. For the year 2020, periods without restrictions on freedom of movement and social contact were compared with periods of moderate and severe COVID-19 restrictions. To avoid distortions due to seasonal fluctuations and linear time trends, suicide risk during the COVID-19 pandemic was compared with data from 2010 to 2019 using an interrupted time series analysis.

**Results:**

A total of 643 suicides were registered and 6 032 690 LY were spent between 2010 and 2020. Of these, 53 suicides and 450 429 LY accounted for the year 2020.

In 2020, SR (suicides per 100 000 LY) were lower in periods with severe COVID-19 restrictions (SR = 7.2, *χ*^2^ = 4.033, *p* = 0.045) compared with periods without restrictions (SR = 16.8). A comparison with previous years showed that this difference was caused by unusually high SR before the imposition of restrictions, while SR during the pandemic were within the trend corridor of previous years (expected suicides = 32.3, observed suicides = 35; IRR = 1.084, *p* = 0.682).

**Conclusions:**

SR during COVID-19 pandemic are in line with the trend in previous years. Careful monitoring of SR in the further course of the COVID-19 crisis is urgently needed. The findings have regional reference and should not be over-generalised.

## Introduction

It remains unclear whether the coronavirus disease 2019 (COVID-19) pandemic is having an impact on suicide rates (SR). Some predict that SR will rise, since actions to contain COVID-19, such as social distancing, economic lockdown or the temporary restructuring of the health system, could cause risk factors for suicide to increase (Fitzpatrick *et al*., 2020; Kawohl and Nordt, 2020; Wand *et al*., 2020; McIntyre and Lee, 2020*a*, 2020*b*). Indeed, analyses of previous economic crises have shown that an increase in unemployment was associated with an increase in SR (Nordt *et al*., 2015; Oyesanya *et al*., 2015; Rachiotis *et al*., 2015; Alicandro *et al*., 2019; Huikari *et al*., 2019). According to leading theories of suicide prevention, the loss of social inclusion is a major risk factor for suicide (van Orden *et al*., 2010). However, it has not yet been clarified whether social cohesion decreased during the pandemic as a result of physical distance or increased, as has been observed during other existential threat scenarios (Durkheim, 1867/1951; Wasserman, 1992; Wasserman *et al*., 2020).

While some studies on previous epidemics found an increase in SR in particular age groups (Wasserman, 1992; Cheung *et al*., 2008; Chang *et al*., 2020; Leaune *et al*., 2020; Zortea *et al*., 2020), the evidence base for suicide risk during the COVID-19 pandemic is very limited (Niederkrotenthaler *et al*., 2020). An initial high-quality study showed no indications of an increase in SR in the early phase of the COVID-19 pandemic in Australia (Leske *et al*., 2020) and a study analysing state-level data from Connecticut found a higher proportion of ethnic minorities among suicide cases during the COVID-19 lock-down compared to preceding years (Mitchell and Li, 2020). Studies using indirect measures for suicide risk during the pandemic provided inhomogeneous results (Gratz *et al*., 2020; Halford *et al*., 2020; Knipe *et al*., 2020; Patsali *et al*., 2020; Sinyor *et al*., 2020).

In Germany, as in other countries, significant restrictions were imposed to contain the COVID-19 pandemic with the strongest restrictions coming into force between 22 March and 5 June.

German borders were virtually closed for travel from 16 March onwards (Federal Ministry of the Interior, 2020*a*, 2020*b*, 2020*c*). On 22 March 2020, the German Federal Government and the Länder agreed on a comprehensive restriction of social contacts, which required people to reduce contacts with others (except for members of one's own household) to an absolute minimum (German Federal Government, 2020*a*). By closing educational and child care facilities, religious sites, all cultural facilities, sports and leisure facilities, hotels, gastronomic establishments and most shops, as well as by prohibiting private gatherings, public and social life was shut down (Free State of Saxony, 2020; German Federal Government, 2020b). Between 4 May and 5 June, contact restrictions were extended to persons from two households.

From 6 June 2020 on, the severe restrictions on going out were eased (Sächsische Staatskanzlei, 2020) and as June progressed, extensive freedom of movement within the EU's Schengen Area was gradually restored, but differentiated travel warnings and quarantine regulations following travel remained in place for parts of it until the end of September 2020 (Federal Foreign Office, 2020).

This study investigated suicide trends during the COVID-19 pandemic and the influence of social distancing during the COVID-19 restrictions on SR. We addressed the following hypotheses:

SR increased in the total population under severe COVID-19 restrictions of social contact compared to periods without or with moderate restrictions.

An interrupted time series analysis shows an increase of suicide risk during the COVID-19 pandemic compared with the time period before onset of the pandemic.

## Methods

### Sample and data acquisition

The data on suicides in this study are based on the City of Leipzig's cause of death statistics, and were provided by the responsible health authority for the years 2010−2020. Annual population statistics were provided by the residents' registration office of Leipzig (https://statistik.leipzig.de/statcity/).

In Germany, physicians determine the cause of death and, if it is uncertain, a medical autopsy may be conducted. The health authorities receive the death certificate and the results of potential autopsies in order to compile statistics on the causes of death. At the time of the investigation, all autopsies had been completed and the causes of death for the cases included had been conclusively determined. However, the total number of suicides may change slightly, e.g. if currently missing persons are found dead at a later date.

### Analytical strategy

Data were analysed using the R software version 3.3.1 (R Core Team, 2016), IBM SPSS 25.0 (IBM Corp., 2019) and Microsoft Excel.

The analysis includes all suicides between January 2010 and September 2020.

For the year 2020, days without restrictions on freedom of movement or social contact were aggregated as period nR_2020 (1 January−16 March), those with moderate restrictions as period R1_2020 (travel restrictions; 17−21 March and 6 June−30 September), and those with severe restrictions as period R2_2020 (restrictions on travel, going out and social contact; 22 March−5 June). To compare suicide mortality in 2020 before and during the COVID-19 restrictions, suicide cases were assigned to groups nR_2020, R1_2020 and R2_2020. Corresponding life years (LY) were calculated, according to the length of the periods examined.

LY and suicide events were used to calculate the risk ratios (RR). Differences in suicide risk among groups nR_2020, R1_2020 and R2_2020 were conducted using chi-square tests.

The suicide risk during the COVID-19 pandemic (March 2020−September 2020) was compared with the suicide risk of the pre-COVID-19 period (January 2010−February 2020). Since the SRs in Germany have been declining in recent years (Alicandro *et al*., 2019), an interrupted time series analysis was performed to control for underlying linear trends and seasonal fluctuations. This approach has been used in previous publications (Chang *et al*., 2020).

Due to overdispersion in the data, we performed a negative binomial regression analysis to identify suicide trends in the pre-COVID-19 period, with annual log-transformed population sizes as offset variable. Seasonal effects were adjusted by defining dummy variables for each calendar month. In a second step, expected suicides for each month of the COVID-19 period were calculated based on the identified time trends.

To examine whether SR during the COVID-19 pandemic were out of line with the time trend of previous years, expected and observed suicides of the COVID-19 period were compared by calculating incidence rate ratios and 95% confidence intervals.

#### Ethical considerations

The study was approved by the ethics committee of the medical faculty of the University Hospital Leipzig, Germany (study ID: 272/20-ek) and conducted in accordance with the Declaration of Helsinki. This epidemiological cohort study is based on the death statistics. For methodological reasons, no informed consent can be obtained.

## Results

A total of 6 032 690 LY were spent and 643 suicides were registered during the periods studied. In 2020, 21 suicides (LY: 124 937) were attributed to nR_2020, 23 (LY: 200 556) to R1_2020 and 9 (LY: 124 937) to R2_2020. The SR within the individual periods were 16.8, 11.5, 7.2 in nR_2020, R1_2020, and R2_2020, respectively.

The suicide risk in 2020 was found to be different between nR_2020 and R2_2020 (*χ*^2^ [1; *N* = 249 903] = 4.033, *p* = 0.045). Comparisons of nR_2020 with R1_2020 (*χ*^2^ [1; *N* = 325 537] = 1.253, *p* = 0.263) and R1_2020 and R2_2020 (*χ*^2^ [1; *N* = 325 525] = 1.023, *p* = 0.312) were not significant (see Table 1).

**Table 1.** Suicide risk before and during COVID-19 restrictions

Group	Focus of restrictions	Suicides	LY	SR		P	
nR_2020	No restrictions	21	124 937	16.8	X 0.263		X
R1_2020	Travel restrictions	23	200 556	11.5	X	X 0.312	0.045
R2_2020	Restrictions on going out, social contact, travel	9	124 937	7.2		X	X

Suicide numbers, person years (LY) and suicide rates (SR, suicides per 100 000 LY) during investigated periods of 2020 without, with moderate and severe COVID-19 restrictions.

**Fig. 1.**
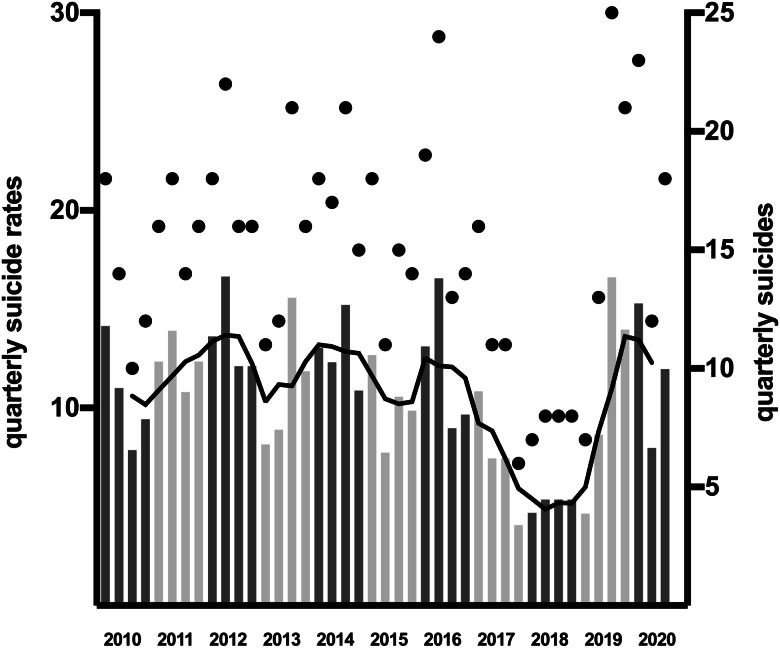
Quarterly suicides and suicide rates from 2010 to 2020. For each quarter of the years 2010 to 2020, the suicide rate (barchart), 12-month moving average (black line) and the number of suicides (black dots) are shown.

**Fig. 2.**
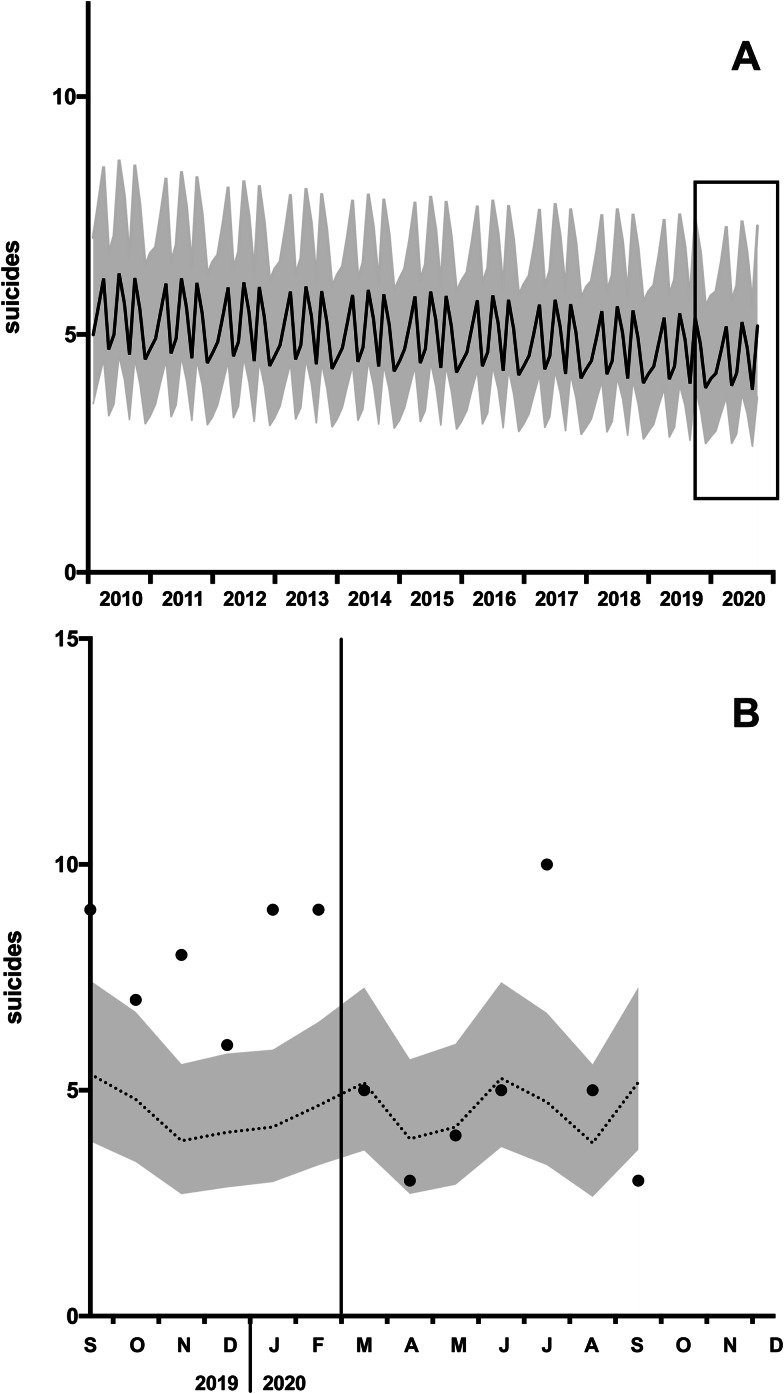
Suicide time trends. (A) Expected suicides (black line) and the 95% confidence interval (95% CI, grey corridor) based on the time series analysis of suicides in 2010 to 2019. The rectangle indicates the section shown below. (B) Projection of expected suicides (doted line), 95%CI and observed suicides (dots) of the individual months.

The time series analysis showed that monthly suicide numbers in the pre-COVID-19 period decreased by 0.3% per month (IRR = 0.997, *p* = 0.028) and the calendar months modulated this linear time trend with an IRR between 0.922 and 1.275 (see Figs 1, 2a). According to the extrapolation of the time trend in 2020, 32.3 expected suicides were calculated for the COVID-19 period March−September 2020 and 35 suicides were observed. The observed versus expected differences were not significant (IRR = 1.084, CI 95% = [0.665, 1.766], *p* = 0.682). Expected and observed suicides of the individual months in 2020 are shown in Fig. 2b.

## Discussion

Our hypotheses regarding SR increasing during COVID-19 restrictions could not be confirmed. The time series analysis revealed that SR during the restrictions were in line with the trend in previous years. In 2020, lower SR were found during severe restrictions compared to the time interval before the restrictions, but this difference was due to unexpectedly high SR in January and February.

The results represent local suicide trends during the COVID-19 pandemic in a major city in Saxony with about 600 000 inhabitants. In Saxony, as in most regions of Germany, the prevalence and mortality rates of COVID-19 were comparatively low during the first wave of the pandemic, with 135.8 and 251.0 cases per 100 000 inhabitants, respectively (Robert Koch Institut, 2020). The regional shut-down was much less restrictive than in other European countries, e.g. United Kingdom, France, Spain and Italy. At an early stage of the pandemic, the German government committed to supporting measures to prevent insolvencies and unemployment. Accordingly, results should be evaluated under these external conditions. Regional differences in the COVID-19 pandemic may produce regional differences in the mental health situation, economic crises and SR. The findings of this study should not, therefore, be extrapolated uncritically to other regions or countries. It should not be assumed either that the trend described will remain stable. This study only provides a first regional snapshot.

The unexpectedly high SR in January and February 2020 are probably unrelated to the pandemic, as they represent a continuation of high SR in 2019 (see Fig. 1). SRs were lowest during the most severe restrictions. Restrictions on going out may act as barriers to outdoor suicide methods, and a person's physical presence at home could strengthen family members' awareness of one another.

Although contact restrictions may contribute to loneliness and a reduced sense of belonging in the medium term, it is suggested that during external threat scenarios, such as war or terrorist attacks, the feeling of social cohesion increases temporarily, and may lead to reduced SR (Durkheim, 1867/1951; Claassen *et al*., 2010). A heightened sense of social cohesion was also registered at the beginning of the COVID-19 pandemic, which found expression in public concern for at-risk groups and the question of how to maintain social inclusion despite the imperative of physical distance (Wasserman *et al*., 2020). However, the study design did not allow us to examine which influencing factors were effective during the restrictions. Future studies are needed to examine how restrictions affect individual risk factors for suicide.

Some studies investigated the association between infectious diseases and suicide risk (Wasserman, 1992; Cheung *et al*., 2008; Chang *et al*., 2020; Leaune *et al*., 2020; Zortea *et al*., 2020). Two studies focused on the impact of severe acute respiratory syndrome on SR in Hong Kong and found higher SR in the elderly (Chan *et al*., 2006; Cheung *et al*., 2008). Two studies found an increase of SR during the influenza pandemic of 1918–1920 in the United States (Wasserman, 1992) and in Taiwan (Chang *et al*., 2020).

There is little evidence of suicide risk during the COVID-19 pandemic. Only one study reports data on SR during the COVID-19 pandemic based on a time series analysis of 3793 suicides in Australia, without any evidence of an increase of suicide risks (Leske *et al*., 2020). A second study analysing state-level data on suicide mortality from Connecticut reported decreasing SR and overrepresentation of ethnic minorities among suicide cases during the COVID 19 quarantine conditions compared with preceding years (Mitchell and Li, 2020).

Projections based on underlying risk factors for suicide, such as unemployment, indicate rising SR during the COVID-19 pandemic (McIntyre and Lee, 2020*a*, 2020*b*). This does not contradict our findings of our study, since although unemployment rose during the COVID-19 pandemic, it remained at a low level during the first months of the pandemic in the region studied (Bundesagentur für Arbeit und Stadt Leipzig, 2020). Our results are consistent with studies reporting no increase in suicidal behaviour, which is used as an indirect measure for suicide risk (Halford *et al*., 2020; Hamm *et al*., 2020; Jacobson *et al*., 2020; Knipe *et al*., 2020; Sakelliadis *et al*., 2020; Sinyor *et al*., 2020; Smalley *et al*., 2020). For instance, online surveys showed a decrease in suicidal thoughts and intention during the pandemic; presentation at emergency departments due to suicidal ideation decreased (Smalley *et al*., 2020), suicides in selective autopsy samples remained low (Sakelliadis *et al*., 2020), and search engine users entered suicide-related terms less frequently (Halford *et al*., 2020; Jacobson *et al*., 2020; Knipe *et al*., 2020; Sinyor *et al*., 2020). However, the overall results are inhomogeneous, since other surveys indicate a high prevalence of suicidal thoughts during the pandemic, in particular under quarantine conditions (Fitzpatrick *et al*., 2020; Gratz *et al*., 2020; Patsali *et al*., 2020).

Overall, there is limited evidence on suicide risk during the COVID-19 pandemic. In many countries, including Germany, national cause-of-death statistics are published with a time lag of several months. During the COVID-19 pandemic, however, up-to-date data are needed to adapt prevention strategies. To facilitate this, the more rapidly available regional data should be published and evaluated in meta-analytical approaches. A platform for this is for example provided by the International COVID-19 suicide prevention research collaboration (ICSPRC) (Gunnell *et al*., 2020; International Association for Suicide Prevention; Niederkrotenthaler *et al*., 2020).

## Conclusion

In the population studied, SR were elevated before onset of COVID-19 restrictions, but there was no increase in SR during restrictions. Careful monitoring of SR as the COVID-19 crisis progresses is essential to establish an evidence base for further prevention approaches. The available results represent a step in this direction.

## Limitations and strengths

This study reports first data on SR during the COVID-19 pandemic in Germany. The study period in 2020 covers nine months and the population studied is relatively small with 0.6 M persons resulting in low suicides numbers. This may result in differences remaining unidentified due to insufficient statistical power.

These findings allow conclusions to be drawn for the region and time period investigated. The results do not allow a supra-regional evaluation or assessment of medium-term trends in SR.

## Data

The paper is based on data provided by the Leipzig Health Authority. These case-related data cannot be published for reasons of data protection. On request, the corresponding author will make aggregated data available to other scientists.

## References

[ref1] Alicandro G, Malvezzi M, Gallus S, La Vecchia C, Negri E and Bertuccio P (2019) Worldwide trends in suicide mortality from 1990 to 2015 with a focus on the global recession time frame. International Journal of Public Health 64, 785–795. 30847527 10.1007/s00038-019-01219-y

[ref2] Appleby L, Arensman E, Hawton K, John A, Kapur N, Khan M, O'Connor RC, Pirkis J, Caine ED, Chan LF, Chang S-S, Chen Y-Y, Christensen H, Dandona R, Eddleston M, Erlangsen A, Harkavy-Friedman J, Kirtley OJ, Knipe D, Konradsen F, Liu S, McManus S, Mehlum L, Miller M, Moran P, Morrissey J, Moutier C, Niederkrotenthaler T, Nordentoft M, O'Neill S, Page A, Phillips MR, Platt S, Pompili M, Qin P, Rezaeian Mohsen, Silverman M, Sinyor M, Stack S, Townsend E, Turecki G, Vijayakumar L and Yip PSF (2020) Suicide risk and prevention during the COVID-19 pandemic. The Lancet Psychiatry 7, 468–471. 32330430 10.1016/S2215-0366(20)30171-1 PMC7173821

[ref3] Bundesagentur für Arbeit und Stadt Leipzig (2020) Available at https://statistik.leipzig.de/statcity/table.aspx?cat=7&rub=3&per=q. https://statistik.leipzig.de/statcity/table.aspx?cat=7&rub=3&per=q (Accessed 8 December 2020).

[ref4] Chan SMS, Chiu FKH, Lam CWL, Leung PYV and Conwell Y (2006) Elderly suicide and the 2003 SARS epidemic in Hong Kong. International Journal of Geriatric Psychiatry 21, 113–118. 16416469 10.1002/gps.1432

[ref5] Chang Y-H, Chang S-S, Hsu C-Y and Gunnell D (2020) Impact of pandemic on suicide: excess suicides in Taiwan during the 1918–1920 influenza pandemic. The Journal of Clinical Psychiatry 81, 20l13454. doi: 10.4088/JCP.20l13454. 32991791

[ref6] Cheung YT, Chau PH and Yip PSF (2008) A revisit on older adults suicides and severe acute respiratory syndrome (SARS) epidemic in Hong Kong. International Journal of Geriatric Psychiatry 23, 1231–1238. 18500689 10.1002/gps.2056

[ref7] Claassen CA, Carmody T, Stewart SM, Bossarte RM, Larkin GL, Woodward WA and Trivedi MH (2010) Effect of 11 September 2001 terrorist attacks in the USA on suicide in areas surrounding the crash sites. The British Journal of Psychiatry: the Journal of Mental Science 196, 359–364. 20435960 10.1192/bjp.bp.109.071928

[ref8] Durkheim E (1867/1951). Suicide: A Study in Sociology. New York: The Free Press.

[ref9] Federal Foreign Office (2020) Außenminister Maas zum Kabinetsbeschluss über die Verlängerung der weltweiten reisewarnung bis zum 31.08. press release. Available at https://www.auswärtiges-amt.de/de/newsroom/weltweite-reisewarnung/2348120.

[ref10] Federal Ministry of the Interior (2020 *a*) Bundesinnenminister Horst Seehofer weitet Binnengrenzkontrollen auf den innereuropäischen Luft- und Seeverkehr aus. Press release of 18.03.2020. Available at https://www.bmi.bund.de/SharedDocs/pressemitteilungen/DE/2020/03/corona-grenzkontrollen-see-luft.html.

[ref11] Federal Ministry of the Interior (2020 *b*) Bundesinnenminister Seehofer ordnet weitreichende Reisebeschränkungen im internationalen Luft- und Seeverkehr an. Press release of 17.03.2020. Available at https://www.bmi.bund.de/SharedDocs/pressemitteilungen/DE/2020/03/corona-reisebeschraenkungen.html.

[ref12] Federal Ministry of the Interior (2020 *c*) Vorübergehende Grenzkontrollen an den Binnengrenzen zu Österreich, der Schweiz, Frankreich, Luxemburg und Dänemark. Press release of 15.03.2020. Available at https://www.bmi.bund.de/SharedDocs/pressemitteilungen/DE/2020/03/grenzschliessung-corona.html.

[ref13] Fitzpatrick KM, Harris C and Drawve G (2020) How bad is it? Suicidality in the middle of the COVID-19 pandemic. Suicide & Life-Threatening Behavior. 10.1111/sltb.12655 [Epub ahead of print]. PMC7361329 32589799

[ref14] Free State of Saxony (2020) Verordnung des Sächsischen Staatsministeriums für Soziales und Gesellschaftlichen Zusammenhalt zum Schutz vor dem Coronavirus SARS-CoV-2 und COVID-19. Sächsische Corona-Schutz-Verordnung – SächsCoronaSchVO. vom 30. April 2020. Sächsisches Gesetz- und Verordnungsblatt, § 10 (1).

[ref15] German Federal Government (2020 *a*) Besprechung der Bundeskanzlerin mit den Regierungschefinnen und Regierungschefs der Länder. Erweiterung der beschlossenen Leitlinien zur Beschränkung sozialer Kontakte. Available at https://www.bundesregierung.de/breg-de/themen/coronavirus/besprechung-der-bundeskanzlerin-mit-den-regierungschefinnen-und-regierungschefs-der-laender-1733248 (Accessed 13 August 2020).

[ref16] German Federal Government (2020 *b*) Besprechung der Bundeskanzlerin mit den Regierungschefinnen und Regierungschefs der Länder am 22. März 2020. Available at https://www.bundesregierung.de/resource/blob/975226/1733246/e6d6ae0e89a7ffea1ebf6f32cf472736/2020-03-22-mpk-data.pdf?download=1.

[ref17] Gratz KL, Tull MT, Richmond JR, Edmonds KA, Scamaldo KM and Rose JP (2020) Thwarted belongingness and perceived burdensomeness explain the associations of COVID-19 social and economic consequences to suicide risk. Suicide & Life-Threatening Behavior. 10.1111/sltb.12654 [Epub ahead of print]. PMC7361587 32589811

[ref18] Halford EA, Lake AM and Gould MS (2020) Google searches for suicide and suicide risk factors in the early stages of the COVID-19 pandemic. PLoS ONE 15, e0236777. 32706835 10.1371/journal.pone.0236777 PMC7380602

[ref19] Hamm ME, Brown PJ, Karp JF, Lenard E, Cameron F, Dawdani A, Lavretsky H, Miller JP, Mulsant BH, Pham VyT, Reynolds CF, Roose SP and Lenze EJ (2020) Experiences of American older adults with pre-existing depression during the beginnings of the COVID-19 pandemic: a multicity, mixed-methods study. The American Journal of Geriatric Psychiatry: Official Journal of the American Association for Geriatric Psychiatry 28, 924–932. 32682619 10.1016/j.jagp.2020.06.013 PMC7305766

[ref20] Huikari S, Miettunen J and Korhonen M (2019) Economic crises and suicides between 1970 and 2011: time trend study in 21 developed countries. Journal of Epidemiology and Community Health 73, 311–316. 30692149 10.1136/jech-2018-210781

[ref21] IBM Corp (2019) IBM SPSS Statistics for Windows. Armonk, NY: IBM Corp.

[ref22] International Association for Suicide Prevention. International COVID-19 Suicide Prevention Research Collaboration (ICSPC). (ICSPRC, Available at https://www.iasp.info/COVID-19_suicide_research.php).

[ref23] Jacobson NC, Lekkas D, Price G, Heinz MV, Song M, O'Malley AJ and Barr PJ (2020) Flattening the mental health curve: COVID-19 stay-at-home orders are associated with alterations in mental health search behavior in the United States. JMIR Mental Health 7, e19347. 32459186 10.2196/19347 PMC7265799

[ref24] Kawohl W and Nordt C (2020) COVID-19, unemployment, and suicide. The Lancet Psychiatry 7, 389–390. 32353269 10.1016/S2215-0366(20)30141-3 PMC7185950

[ref25] Knipe D, Evans H, Marchant A, Gunnell D and John A (2020) Mapping population mental health concerns related to COVID-19 and the consequences of physical distancing: a Google trends analysis. Wellcome Open Research 5, 82. 32671230 10.12688/wellcomeopenres.15870.1 PMC7331103

[ref26] Leaune E, Samuel M, Oh H, Poulet E and Brunelin J (2020) Suicidal behaviors and ideation during emerging viral disease outbreaks before the COVID-19 pandemic: a systematic rapid review. Preventive Medicine 141, 106264. 33017599 10.1016/j.ypmed.2020.106264 PMC7531915

[ref27] Leske S, Kõlves K, Crompton D, Arensman E and de Leo D (2020) Real-time suicide mortality data from police reports in Queensland, Australia, during the COVID-19 pandemic: an interrupted time-series analysis. The Lancet. Psychiatry 8, 58–63. 33212023 10.1016/S2215-0366(20)30435-1 PMC7836943

[ref28] McIntyre RS and Lee Y (2020 *a*) Preventing suicide in the context of the COVID-19 pandemic. World Psychiatry : Official Journal of the World Psychiatric Association *(*WPA*)* 19, 250–251. 32394579 10.1002/wps.20767 PMC7214950

[ref29] McIntyre RS and Lee Y (2020 *b*) Projected increases in suicide in Canada as a consequence of COVID-19. Psychiatry Research 290, 113104. 32460184 10.1016/j.psychres.2020.113104 PMC7236718

[ref30] Mitchell TO and Li L (2020) State-level data on suicide mortality during COVID-19 quarantine: early evidence of a disproportionate impact on racial minorities. Psychiatry Research 295, 113629. 33290944 10.1016/j.psychres.2020.113629

[ref31] Niederkrotenthaler T, Gunnell D, Arensman E, Pirkis J, Appleby L, Hawton K, John A, Kapur N, Khan M, O'Connor RC and Platt S (2020) Suicide research, prevention, and COVID-19. Crisis 41, 321–330. 32716205 10.1027/0227-5910/a000731 PMC8729451

[ref32] Nordt C, Warnke I, Seifritz E and Kawohl W (2015) Modelling suicide and unemployment: a longitudinal analysis covering 63 countries, 2000–2011. The Lancet. Psychiatry 2, 239–245. 26359902 10.1016/S2215-0366(14)00118-7

[ref33] Oyesanya M, Lopez-Morinigo J and Dutta R (2015) Systematic review of suicide in economic recession. World Journal of Psychiatry 5, 243–254. 26110126 10.5498/wjp.v5.i2.243 PMC4473496

[ref34] Patsali ME, Mousa D-PV, Papadopoulou EVK, Konstantina KK, Kaparounaki CK, Diakogiannis I and Konstantinos N (2020) University students’ changes in mental health status and determinants of behavior during the COVID-19 lockdown in Greece. Psychiatry Research 292, 113298. 32717710 10.1016/j.psychres.2020.113298 PMC7357537

[ref35] Rachiotis G, Stuckler D, McKee M and Hadjichristodoulou C (2015) What has happened to suicides during the Greek economic crisis? Findings from an ecological study of suicides and their determinants (2003–2012). *BMJ* Open 5, e007295. 10.1136/bmjopen-2014-007295 PMC4386238 25807950

[ref36] R Core Team (2016) R: A Language and Environment for Statistical Computing. Vienna, Austria: R Foundation for Statistical Computing. Available at https://www.R-project.org.

[ref37] Robert Koch Institut (2020) Coronavirus SARS-CoV-2. Daily situation reports in German and English (31.07.2020). Available at https://www.rki.de/DE/Content/InfAZ/N/Neuartiges_Coronavirus/Fallzahlen.html.

[ref38] Sächsische Staatskanzlei (2020) Verordnung des Sächsischen Staatsministeriums für Soziales und Gesellschaftlichen Zusammenhalt zum Schutz vor dem Coronavirus SARS-CoV-2 und COVID-19 (Sächsische Corona-Schutz-Verordnung – SächsCoronaSchVO) vom 3. Juni 2020. Available at https://www.laenderrecht.de/media/upload/0227%20-%20SaechsGVBl_2020-12_LV.pdf#page=2.

[ref39] Sakelliadis EI, Katsos KD, Zouzia EI, Spiliopoulou CA and Tsiodras S (2020) Impact of Covid-19 lockdown on characteristics of autopsy cases in Greece. Comparison between 2019 and 2020. *Forensic* Science International 313, 110365. 32563134 10.1016/j.forsciint.2020.110365 PMC7291972

[ref40] Sinyor M, Spittal MJ and Niederkrotenthaler T (2020) Changes in suicide and resilience-related Google searches during the early stages of the COVID-19 pandemic. Canadian Journal of Psychiatry. Revue canadienne de psychiatrie 65, 741–743. 32524848 10.1177/0706743720933426 PMC7502879

[ref41] Smalley CM, Malone DA, Meldon SW, Borden BL, Simon EL, Muir MR and Fertel BS (2020) The impact of COVID-19 on suicidal ideation and alcohol presentations to emergency departments in a large healthcare system. The American Journal of Emergency Medicine. 10.1016/j.ajem.2020.05.093 [Epub ahead of print]. PMC7263212 32505472

[ref42] van Orden KA, Witte TK, Cukrowicz KC, Braithwaite SR, Selby EA and Joiner TE (2010) The interpersonal theory of suicide. Psychological Review 117, 575–600. 20438238 10.1037/a0018697 PMC3130348

[ref43] Wand APF, Zhong B-L, Chiu HFK, Draper B and de Leo D (2020) COVID-19: the implications for suicide in older adults. International Psychogeriatrics 32, 1225–1230. 32349837 10.1017/S1041610220000770 PMC7235297

[ref44] Wasserman IM (1992) The impact of epidemic, war, prohibition and media on suicide: United States, 1910-1920. Suicide & Life-Threatening Behavior 22, 240–254. 1626335

[ref45] Wasserman D, van der Gaag R and Wise J (2020) The term “physical distancing” is recommended rather than “social distancing” during the COVID-19 pandemic for reducing feelings of rejection among people with mental health problems. European Psychiatry 63, e52. 32475365 10.1192/j.eurpsy.2020.60 PMC7287304

[ref46] Zortea TC, Brenna CTA, Joyce M, McClelland H, Tippett M, Maxwell M, Arensman E, Corcoran P, Hatcher S, Heise MJ, Links P, O'Connor R, Edgar NE, Cha Y, Guaiana G, Williamson E, Sinvor M and Platt S (2020) The impact of infectious disease-related public health emergencies on suicide, suicidal behavior, and suicidal thoughts. Crisis, 1–14. 10.1027/0227-5910/a000753 PMC8689932 33063542

